# PD95 Enhancing Clinical Practice Guideline Development In Brazil: A Tutoring Program For Health Technology Assessment Centers

**DOI:** 10.1017/S0266462324003428

**Published:** 2025-01-07

**Authors:** Maicon Falavigna, Cintia Araujo, Suena Parahiba, Gilson Dorneles, Cinara Stein

## Abstract

**Introduction:**

This study outlines a tutoring program supported by the Brazilian Ministry of Health (MoH), which aimed to develop clinical practice guidelines (CPGs) through Health Technology Assessment Centers (Nuclei of Health Technology Assessment or NATS) for the Brazilian Unified Health System (SUS). It emphasizes the integration of CPGs into health technology assessment development processes, focusing on methodological rigor, reproducibility, and reliability.

**Methods:**

The program combined face-to-face and virtual meetings and engaged MoH representatives, methodologists, and researchers. It focused on the MoH Methodological Guideline for Developing Clinical Guidelines, which follows the Guidelines International Network and United States Institute of Medicine recommendations. This approach facilitated planning and creation of CPGs across diverse NATS.

**Results:**

Between 2021 and 2023, 60 professionals from 10 NATS participated, aiding in developing or updating 16 CPGs. These CPGs addressed 93 research questions. The CPG development phase averaged 317 days (interquartile range [IQR] 252 to 402), while the MoH assessment and public consultation took about 63 days (IQR 45 to 94). Additionally, nine Protocolos Clínicos e Diretrizes Terapêuticas (official guidelines from the Brazilian MoH) required technology assessments for SUS reimbursement, leading to 14 HTA reports. Eight technologies were favorably reviewed and recommended in the CPGs.

**Conclusions:**

This tutoring program significantly improved the development of CPGs in Brazil, enhancing their methodological rigor and standardization. It also effectively integrated HTA into the CPG development process, addressing clinical needs and enriching public health decision-making.

